# Clinical outcomes of nicorandil administration in patients with acute ST-segment elevation myocardial infarction undergoing primary percutaneous coronary intervention: a systematic review and meta-analysis of randomized controlled trials

**DOI:** 10.1186/s12872-021-02301-1

**Published:** 2021-10-10

**Authors:** Ning Geng, Li Ren, Lisheng Xu, Deling Zou, Wenyue Pang

**Affiliations:** 1 Department of Cardiology, Shengjing Hospital of China Medical University, No. 36 Sanhao Street, Heping District, Shenyang City, Liaoning Province China; 2 Sino-Dutch Biomedical and Information Engineering School, Northeastern University (Hunnan Campus), No. 195, Chuangxin Street, Hunnan District, Shenyang City, Liaoning Province China

**Keywords:** Nicorandil, ST-segment elevation myocardial infarction, Primary percutaneous coronary intervention, No-reflow phenomenon, major adverse cardiovascular event

## Abstract

**Background:**

Primary percutaneous coronary intervention is the treatment of choice in ST-segment elevation myocardial infarction and no-reflow phenomenon is still an unsolved problem.

**Methods:**

We searched PubMed, EmBase, and Cochrane Central Register of Controlled Trials for relevant randomized controlled trials. The primary endpoint was the incidence of major adverse cardiac events and the secondary endpoint was the incidences of no-reflow phenomenon and complete resolution of ST-segment elevation.

**Results:**

Eighteen randomized controlled trials were enrolled. Nicorandil significantly reduced the incidence of no-reflow phenomenon (OR, 0.46; 95% CI, 0.36–0.59; P < 0.001; I^2^ = 0%) and major adverse cardiac events (OR, 0.42; 95% CI, 0.27–0.64; P < 0.001; I^2^ = 52%). For every single outcome of major adverse cardiac events, only heart failure and ventricular arrhythmia were significantly improved with no heterogeneity (OR, 0.36; 95% CI, 0.23–0.57, P < 0.001; OR, 0.43; 95% CI, 0.31–0.60, P < 0.001 respectively). A combination of intracoronary and intravenous nicorandil administration significantly reduced the incidence of major adverse cardiac events with no heterogeneity (OR, 0.24; 95% CI, 0.13–0.43, P < 0.001; I^2^ = 0%), while a single intravenous administration could not (OR, 0.66; 95% CI, 0.40–1.06, P = 0.09; I^2^ = 52%).

**Conclusions:**

Nicorandil can significantly improve no-reflow phenomenon and major adverse cardiac events in patients undergoing primary percutaneous coronary intervention. The beneficial effects on major adverse cardiac events might be driven by the improvements of heart failure and ventricular arrhythmia. A combination of intracoronary and intravenous administration might be an optimal usage of nicorandil.

**Supplementary Information:**

The online version contains supplementary material available at 10.1186/s12872-021-02301-1.

## Background

Primary percutaneous coronary intervention (PCI) has been the treatment of choice for ST-segment elevation myocardial infarction (STEMI) in the era of reperfusion. Although door-to-balloon times improve significantly, in-hospital mortality has remained virtually unchanged [1]. Re-opening of an infarct-related coronary artery by primary PCI does not always restore myocardial perfusion, due to the no-reflow phenomenon (NRP) in up to 30% of patients, which is associated with larger infarct size and worse outcomes [2]. Distal microthrombus embolization after balloon dilation or stent implantation and microvascular damages caused by ischemia–reperfusion injury play important roles in the genesis of NRP [3].

Nicorandil, a hybrid of the adenosine triphosphate-sensitive potassium (K^+^_ATP_) channel opener and nitrate, can not only cause vasodilation of both the epicardial coronary arteries and coronary resistance arterioles, but also exert pharmacological preconditioning effects by opening mitochondrial K^+^_ATP_ channel [4]. So nicorandil might improve NRP and clinical outcomes after primary PCI in patients with STEMI by ameliorating ischemia–reperfusion injury and improving microvascular function. The administration of intravenous nicorandil was recommended as a Class IIb, Level of Evidence B therapy for patients undergoing primary PCI in the 2018 JCS (Japanese Circulation Society) guideline on acute coronary syndrome [5]. Several randomized controlled trials (RCTs) have been conducted to evaluate the clinical efficacy of nicorandil in patients treated by primary PCI and significantly heterogeneous clinical outcomes have been achieved, though improved microvascular function was guaranteed. The trial conducted by Feng et al. had shown that nicorandil could reduce the rate of NRP after primary PCI and improve the clinical outcomes at 6 months follow-up [6]. However, these better effects were not demonstrated in some other trials. Chen et al. investigated the effects of intracoronary nicorandil in individuals undergoing primary PCI for acute inferior myocardial infarction [7]. No significantly reduced no-reflow rate (OR: 0.57, 95% CI: 0.22–1.46) and major adverse cardiac events (MACE) (OR: 0.48, 95% CI: 0.08–2.74) were found in patients with the use of nicorandil. There were also great heterogeneities in the usage of nicorandil (intracoronary, intravenous or both during primary PCI, with/without following intravenous nicorandil after primary PCI). To clarify the effects of nicorandil administration on NRP, clinical outcomes and explore the optimal usage of nicorandil in patients undergoing primary PCI, we performed this systematic review and meta-analysis.

## Methods

We conducted this study in accordance with the preferred reporting items for systematic reviews and meta-analysis (PRISMA) checklist [8]. This study is a meta-analysis of RCTs and all data were collected from published trials, so an additional ethical approval is not necessary.

### Literature search

We searched PubMed, Embase, and Cochrane Central Register of Controlled Trials with no language restriction for relevant articles till May 8, 2021 by the PICOS search strategy. Combinations of MeSH terms, entry terms, and text words were used for the search of every theme. For the theme ‘nicorandil’, we used the following key words Nicorandil OR 2-Nicotinamidoethyl Nitrate OR SG 75 OR Ikorel OR Adancor OR Dancor. For the theme ‘ST elevation myocardial infarction’, we used: ST Elevation Myocardial Infarction OR ST Segment Elevation Myocardial Infarction OR ST Elevated Myocardial Infarction OR STEMI. Randomized controlled trial OR controlled clinical trial OR randomized OR randomly were used for the theme ‘randomized controlled trial’. For the final search results, we combined the search results of each theme by the Boolean operator ‘AND’. We also performed manual search for potential eligible studies. Authors of published studies were also contacted for more data as needed. For studies of overlapping patient populations, data from the most informative or most recent publications were included in our meta-analysis.

### Eligibility criteria

The inclusion criteria were as follows: RCTs; adult patients with STEMI undergoing primary PCI; nicorandil with/without other positive drugs were administrated intravenously or via intracoronary in experimental groups, while placebo with/without other positive drugs were given in control groups; the clinical outcomes and/or myocardial reperfusion measurements such as thrombolysis in myocardial infarction (TIMI) flow grade; TIMI myocardial perfusion grade (TMPG); complete resolution of ST-segment elevation (STR) on ECG after primary PCI were reported in both experimental and control arms. The followings were excluded: conference abstracts without needed data; no clinical outcomes and incidences of NRP were reported; only oral nicorandil was administrated.

### Data extraction

Two reviewers (NG and DLZ) independently extracted data from all eligible studies. Disagreements were resolved through discussion with all the reviewers. The extracted information included: first author; year of publication; sample size; patient characteristics; procedure; interventions in treatment and control groups; dosage and administration method of nicorandil; incidences of MACEs, NRP and complete STR. If enrolled studies included more than 2 arms, we combined the arms in which nicorandil was administrated as experimental groups; arms with no nicorandil administration as control groups. For a binary outcome (incidences of MACEs, NRP or complete STR), combining the arms simply means adding the numbers of events and total participants over all arms. The clinical results at the longest follow-up durations were employed for the pooled analyses to maximize the effects of nicorandil if clinical results were reported at different follow-up durations.

### Assessment of risk of bias

Two reviewers (NG and LR) independently assessed risk of bias for each eligible study by creating risk of bias graph and risk of bias summary graph, using the Cochrane Collaboration’s tool for assessing risk of bias. This tool evaluated each trial by considering the following sources of bias: selection bias; performance bias; attrition bias; detection bias; reporting bias; and other potential sources of bias. The risk of each bias was evaluated and rated as “low,” “unclear,” or “high”. Any discrepancy was solved by discussion.

### Methods of assessing clinical NRP

Several measurements were applied for the evaluation of NRP. We chose TMPG as the measurement of choice. TIMI flow grade would be used if TMPG was not reported. Other measurements, such as corrected TIMI frame count (cTFC) and myocardial contrast echocardiography (MCE), would be employed depending on the author’s choice if neither TMPG nor TIMI flow grade were reported.

### Statistical analyses

The primary endpoint of our meta-analysis was the incidence of MACE and the secondary endpoint was the incidences of NRP and complete STR. Statistical analyses of effect sizes were performed by Review Manager 5.3 (The Cochrane Collaboration, Copenhagen, DK). Odds ratio (OR) and 95% confidence interval (CI) were used to describe dichotomous data (incidences of MACE, NRP, complete STR) for each study. The heterogeneity across trials was quantified using the I^2^ statistic, which indicates the percentage of total variation attributed to statistical heterogeneity rather than chance, with I^2^ < 25%, 25% to 50%, and > 50% representing mild, moderate, and severe heterogeneity respectively [9]. Subgroup analyses were also conducted to explore the origin of heterogeneity; optimal usage of nicorandil; efficacy of nicorandil with/without following intravenous administration after primary PCI procedure; efficacy of nicorandil at different follow-up durations. We pooled the trials using random-effects model and estimated the absolute between-study variance using the DerSimonian and Laird estimator, considering the potential heterogeneities across included trials due to expected clinical and methodological heterogeneities that might manifest as statistical heterogeneity.

The test of publication bias was performed by CMA 3.0. We used Egger's regression to test the symmetricity of the funnel plot; and Trim and Fill approach to assess the impact of potential publication bias by estimating what studies might be missed and then imputing them to the pooled analysis.

Sensitivity analysis was performed to assess the stability of the results by removing a single trial in turn and pooling the remaining ones. For all of the results, intention-to-treat analyses were utilized. *P* < 0.05 in 2-tailed tests was considered statistically significant.

## Results

### Study selection

**Fig. 1:**
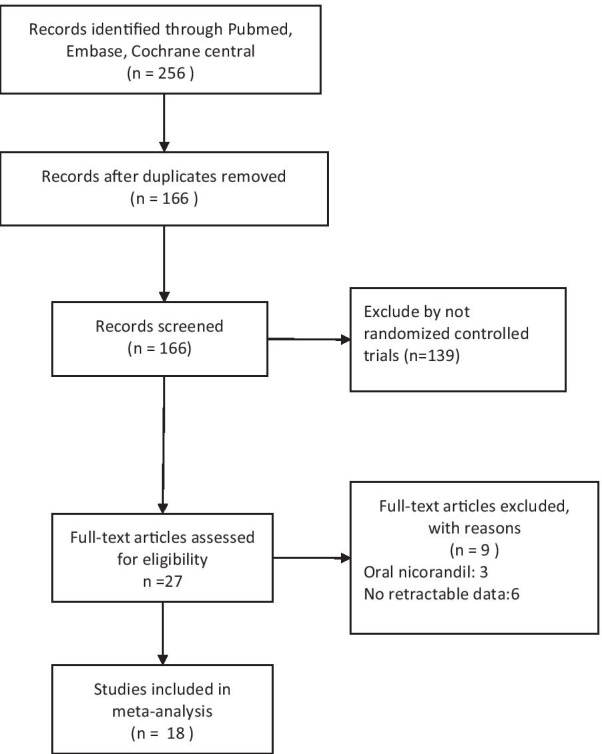
Flow chart for study selection

We identified 256 potentially relevant citations from the initial search. After removing the duplicates and screening the titles and abstracts, 27 full-text articles were deemed to be assessed for eligibility. No clinical outcomes and NRP were reported in 6 trials and only oral nicorandil was administrated in 3 trials. Therefore, 18 RCTs [6, 7, 10–25] involving 2398 patients with STEMI undergoing primary PCI were identified and analyzed. Our search strategy and results were outlined in Fig. 1.

### Characteristics of included studies

Characteristics of included studies are presented in Table 1. Only intravenous nicorandil was administrated in 6 trials [10, 12–14, 18, 25]; only intracoronary nicorandil in 5 trials [6, 7, 15, 21, 22]; both intravenous and intracoronary nicorandil in 7 trials [11, 16, 17, 19, 20, 23, 24] during primary PCI. Oral nicorandil was administrated during follow-up in 2 trials [13, 16]. Six studies had more than 2 groups (5 studies: 3 groups [17, 19–22]; 1 study: 4 groups [7]).

**Table 1:** Characteristics of included studies

Study	Year	Onset to reperfusion time, (hours). (N/C)	Sample size (N/C)	Interventions		Followup
				Nicorandil	Control	
Chen et al. [6]	2015	7.1/7.0	52/52	2 mg ic or 2 mg + anisodamine 2 mg ic	No treatment or anisodamine 2 mg i.c	IH, 30d
Chen et al. [23]	2020	NA	39/39	0.06 mg/kg ic; 2 mg/h iv for 36 h tirofiban 10 μg/kg ic; 0.1 μg/kg·m iv for 36 h	Tirofiban 10 μg/kg ic 0.1 μg/kg·m iv for 36 h	IH, 30d
Feng et al. [5]	2019	4.7/4.8	90/90	2–6 mg ic; thrombectomy; Tirofiban 10 mg/kg ic	Saline 2–6 mL i.c.; thrombectomy; Tirofiban 10 mg/kg i.c	1,3,6 m
Fukuzawa et al. [9]	2000	4.6/4.5	31/31	4 mg bolus iv, 6 mg/h iv for 24 h	No agent	IH
Ikeda et al. [10]	2004	5.2/5.7	30/30	6 mg/h iv for 72 h, 2 mg ic	Isosorbide dinitrate 6 mg/h iv for 72 h, 2 mg ic	IH
Ishii et al. [11]	2005	4.8/4.5	185/183	12 mg + saline 100 ml iv isosorbide 2.5-5 mg ic	Saline 100 ml i.v.; isosorbide 2.5-5 mg i.c	2.4y*
Ito et al. [12]	1999	4.8/5.3	40/41	4 mg bolus iv; 6 mg/h for 24 h 15 mg/day po (a mean of 28 days)	No agent	IH
Kitakaze et al. [13]	2007	4.20/4.25	276/269	0.067 mg/kg bolus iv 1∙67 μg/kg.min iv for 24 h	Saline by the same method	2.5y*
Lee et al. [14]	2008	5.9/5.8	37/36	2 mg ic before CAG 2 mg ic before stenting	No agent	IH;30d
Miyazawa et al. [15]	2006	6.1/8.0	35/35	2 mg ic distal to lesion; 2 mg/h for 24hiv; 15 mg/d po	No agent	8 m
Nameki et al. [16]	2004	5.85/6.17	13/27	4 mg iv 4 mg ic before reperfusion, 4 mg/h iv for 24 h	Magnesium: 10 mmol iv before reperfusion, 0.4 mmol/h for 24 h or no agent	IH;3 m
Ono et al. [17]	2004	5.6/5.1	33/25	4 mg bolus iv, 8 mg/h iv for 24 h after PCI	No agent	IH;6 m
Ota et al. [18]	2006	4.05/3.86	63/27	1–2 mg ic or 1–2 mg ic + 4 mg bolus iv, 6 mg/h iv(total:96 mg)	No agent	IH
Pi et al. [19]	2019	6.51/6.86	95/45	a:4 mg ic, 4 mg/h iv for 24 h b:4 mg ic,saline 4 ml/h iv for 24 h	Saline:8 ml ic, 4 ml/h iv for 24 h	IH
Qi et al. [20]	2018	5.7/6.1	40/80	2 mg ic	Nitroprusside:200 μg ic or saline only	IH;3 m
Wang et al. [21]	2017	4.8/4.5/3.8*	53/105	6 mg ic	NG 300 μg ic or no agent	IH;3 m
Wang et al. [24]	2020	5.9/5.7	59/60	iv, dosage not mentioned	No agent	6 m
Yamada et al. [22]	2015	6.4/6.8	28/24	0.2 mg/kg ic before the initial and final angiograms;2.0 mg/h iv for 4 days	Nitroglycerin 0.2 mg/kg ic before the initial and final angiograms;2.0 mg/h iv for 4 days	IH

Ic, intracoronary; IH, in hospital; iv, intravenous; N/C, nicorandil/control groups; po, per oral

*Median

### Patient characteristics

The major characteristics of the patients in every enrolled trial are shown in Additional file 6: Table 1. All the baseline characteristics (age, gender, diabetes, hypertension) were statistically similar between the experimental groups and control groups in each trial.

### Risks of bias within studies

Risk of bias graph and risk of bias summary graph are presented in Additional file 1: Fig. 1 and Additional file 2: Fig. 2 separately, which evaluated the relevant study characteristics according to Cochrane Handbook for Systematic Reviews of Interventions. Only 6 trials [6, 14, 20, 22, 24, 25] reported methods of random sequence generation and 4 trials [12, 14, 18, 19] described the concealments of allocation.

**Fig. 2:**
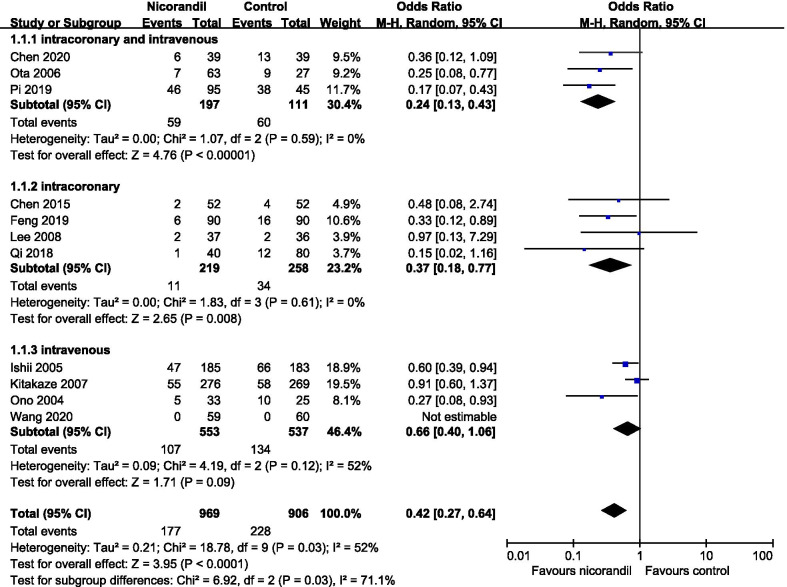
Subgroup analysis of major adverse cardiovascular events based on different methods of nicorandil administration

### Major adverse cardiac events

MACEs were defined as a combination of mortality, new onset of acute myocardial infarction (AMI), target vessel revascularization (TVR), re-hospitalization for congestive heart failure (CHF) and ventricular arrhythmia (ventricular tachycardia or fibrillation). The MACEs were predefined and reported in 9 studies [6, 7, 14, 15, 18–21, 24]. In study by Ishii et al. [12] and Wang et al. [25], the MACEs were not pre-defined, but composite end points of all-cause mortality, all-cause re-admission (Re-PCI; CABG) were reported in study by Ishii et al.; all-cause death, cardiovascular death, unplanned hospitalization for CHF, TVR in study by Wang et al. We defined these composite end points as MACEs in these 2 studies respectively. So, 11 studies were used for the pooled analysis of MACEs. Nicorandil was administrated in 969 patients, whereas 906 patients were in control groups. The overall incidence of MACEs in nicorandil groups was 18.3% compared with 25.2% in the control groups. Nicorandil did significantly reduce the incidence of MACEs, but a severe heterogeneity existed (OR, 0.42; 95% CI, 0.27–0.64; P < 0.001; I^2^ = 52%; Fig. 2).

To detect the origin of the severe heterogeneity and clinical effects produced by different methods of nicorandil administration, we performed a subgroup analysis based on the administrating methods of nicorandil (intracoronary plus intravenous vs. intracoronary vs. intravenous). The results were shown in Fig. 2. A combination of intracoronary and intravenous nicorandil was administrated in 3 studies [19, 20, 24]; intracoronary nicorandil in 4 studies [6, 7, 15, 21] and intravenous nicorandil in 4 studies [12, 14, 18, 25]. No heterogeneities were present in the combination and the intracoronary subgroups (combination subgroup: Tau^2^ = 0.00; Chi^2^ = 1.07, P = 0.59; I^2^ = 0%; intracoronary subgroup: Tau^2^ = 0.00; Chi^2^ = 1.83, P = 0.61; I^2^ = 0%). There was a still severe heterogeneity in intravenous subgroup (Tau^2^ = 0.09; Chi^2^ = 4.19, P = 0.12; I^2^ = 52%). So, the different methods of nicorandil usage might act as a partial origin of heterogeneity. We found a trend of less risk of MACEs across the intravenous, intracoronary and intracoronary plus intravenous subgroups (OR: 0.66; 0.37; 0.24 respectively). Interestingly a single intracoronary administration or combined with intravenous administration could significantly reduce the incidence of MACEs (intracoronary subgroup: OR, 0.37; 95% CI, 0.18–0.77; P = 0.008; combination subgroup: OR, 0.24; 95% CI, 0.13–0.43; P < 0.001), but a single intravenous administration could not (OR, 0.66; 95% CI, 0.40–1.06; P = 0.09). Combination of intracoronary and intravenous nicorandil had a significantly lower incidence of MACEs compared with a single intravenous nicorandil (Chi^2^, 6.76; I^2^, 85.2%; P_interaction_ = 0.009, data not shown). So, a single intravenous administration might not be an optimal usage of nicorandil, while a combination of intracoronary and intravenous nicorandil might be.

In 7 studies [6, 7, 12, 15, 19–21], intracoronary and/or intravenous nicorandil were administrated only during the primary PCI procedures in the experimental groups or one arm of the experimental groups (we defined these studies as ‘no nicorandil after PPCI’ subgroup). While in 5 studies [14, 18–20, 24], intracoronary and/or intravenous nicorandil were followed by a continuous intravenous nicorandil infusion after the procedure in the experimental groups or one arm of the experimental groups (we defined these studies as ‘maintaining nicorandil after PPCI’ subgroup). In order to explore the effects of continuous maintaining nicorandil after primary PCI on clinical outcomes, we performed a subgroup analysis comparing the clinical outcomes between the 2 subgroups. Nicorandil can significantly reduce the incidence of MACEs in both subgroups. We found a trend of less risk of MACEs in ‘maintaining nicorandil after PPCI’ subgroup (OR, 0.30; 95% CI, 0.12–0.79) compared with ‘no nicorandil after PPCI’ subgroup (OR, 0.47; 95% CI, 0.33–0.67) (Additional file 3: Fig. 3). But the difference was not statistically significant (I^2^ = 0%, P_interaction_ = 0.40). The additional continuous intravenous nicorandil infusion did not further reduce the incidence of MACEs significantly, which further suggested that intravenous nicorandil administration might not be an optimal usage.

**Fig. 3:**
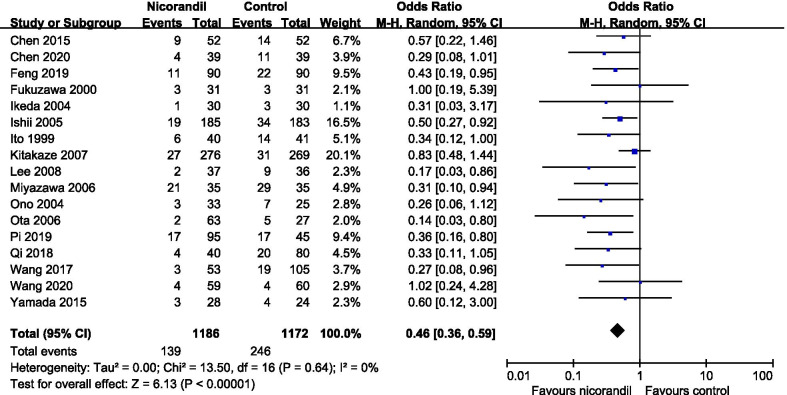
Forest plot of no-reflow phenomena

Some of the included studies only reported the MACEs during in-hospital stay [18–20], while some others followed patients for more than two years [12, 14]. We performed a subgroup meta-analysis to evaluate the MACEs during in-hospital stay and during follow-up after hospital discharge. In-hospital or follow-up MACEs were reported in 6 [7, 15, 18–21] and 7 [6, 7, 12, 14, 15, 21, 24] studies (range from 1 month to 2.5 years) respectively. We found that the risk of MACEs could be significantly reduced during in-hospital (OR,0.23; 95% CI, 0.13 to 0.41; P < 0.001) and the beneficial effect could be maintained during the follow-up (OR,0.60; 95% CI, 0.42 to 0.86; P = 0.006), (Additional file 4: Fig. 4). There were no and mild heterogeneities in these 2 subgroups respectively (In-hospital subgroup: I^2^ = 0%; follow-up subgroup: I^2^ = 23%). So, the different follow-up durations might also be an origin of heterogeneity.

**Fig. 4:**
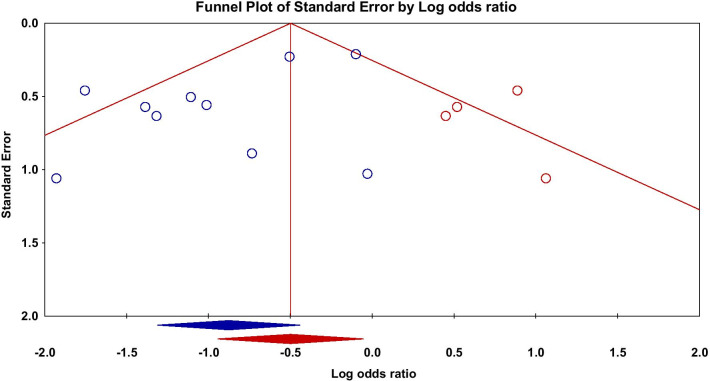
Funnel plot of major adverse cardiovascular events. blue diamond, odds ratio and 95% confidence interval of pooled major adverse cardiovascular events; red circles, assumed missing studies; red diamond, adjusted odds ratio and 95% confidence interval by Trim and Fill method after imputing 4 assumed missing studies to the pooled analysis

### Meta-analyses on every single outcome of MACEs

In order to explore the effects of nicorandil on every single outcome of MACEs, we also performed pooled analyses on mortality [6, 7, 12–17, 20–22, 24, 25]; new-onset AMI [7, 15, 16, 18, 21]; TVR [7, 12, 15, 16, 20–22, 25]; re-hospitalization for CHF [6, 12, 13, 16, 18, 20–23, 25]; arrhythmia (ventricular tachycardia or fibrillation) [11–13, 17–22, 25] separately. The results were presented in Table 2. The nicorandil administration did reduce the incidences of re-hospitalization for CHF (OR, 0.36; 95% CI, 0.23–0.57; P < 0.001) and ventricular arrhythmia (OR, 0.43; 95% CI, 0.31–0.60; P < 0.001) with no heterogeneity (CHF: Tau^2^ = 0.00; P = 0.55; I^2^ = 0%; ventricular arrhythmia: Tau^2^ = 0.00; P = 0.91; I^2^ = 0%), but not the mortality (OR, 0.68; 95% CI, 0.41–1.11; P = 0.12; I^2^ = 0%), new AMI (OR, 0.56; 95% CI, 0.19–1.67; P = 0.30; I^2^ = 0%), TVR (OR, 1.01; 95% CI, 0.64–1.59; P = 0.95; I^2^ = 0%). So, the improvement of MACEs was mainly driven by the favorable effects on CHF and ventricular arrhythmia. Severe heterogeneity existed in the pooled analysis of MACEs but disappeared when every single outcome was pool-analyzed. The different predefinitions of MACEs among the included studies might be an important origin of the severe heterogeneity.

**Table 2:** Meta-analyses of mortality, new AMI, TVR, heart Failure, arrhythmia

Clinical outcomes	Studies included	Effect sizes			Heterogeneity		
		OR	95% CI	P value	Tau^2^	I^2^ (%)	P value
Mortality	13	0.68	0.41–1.11	0.12	0.00	0	0.83
New AMI	5	0.56	0.19–1.67	0.30	0.00	0	0.90
TVR	8	1.01	0.64–1.59	0.95	0.00	0	0.77
Heart failure	10	0.36	0.23–0.57	0.00	0.00	0	0.55
Arrhythmia	10	0.43	0.31–0.60	0.00	0.00	0	0.91

Arrhythmia refers to ventricular tachycardia and ventricular fibrillation

AMI, acute myocardial infarction; CI, confidence interval; OR, odds ratio; TVR, target vessel revascularization

### No-reflow phenomenon

A total of 17 studies [6, 7, 10–16, 18–25] reported NRP and were used for pooled analysis. TMPG, TIMI grade, cTFC and MCE were used for pooled analysis in 5 [6, 7, 16, 21, 24], 10 [10–12, 14, 15, 18–20, 23, 25], 1 [22], and 1 [13] studies respectively. The pooled analysis showed that nicorandil administration significantly reduced incidence of NRP with no heterogeneity (OR, 0.46; 95% CI, 0.36 to 0.59; P < 0.001; I^2^ = 0%; Fig. 3).

### Resolution of ST-segment elevation

Complete STR can serve as a simple and practical index of microvascular function and myocardial reperfusion after primary PCI. So, we performed a meta-analysis on complete STR to evaluate nicorandil’s effect on microvascular function. Ten studies [6, 7, 11, 12, 19–22, 24, 25] reported complete STR and were used for the pooled analysis. The pooled result showed a beneficial effect of nicorandil on complete STR with no heterogeneity (OR, 2.86; 95% CI, 2.19 to 3.73; P < 0.001; I^2^ = 0%, Additional file 5: Fig. 5).

### Publication bias

Egger's regression showed a significantly asymmetrical funnel plot of MACE (intercept = − 1.75, P = 0.04). Studies with smaller sample sizes tended to have less MACE risk (Fig. 4). We applied random effects model for our meta-analysis, different studies might represent different populations. So, the possibility of smaller studies really having a greater effect size couldn’t be excluded. In our meta-analysis, studies in combination subgroup with relatively small sample sizes had less risk for MACE than those in intravenous subgroup. So, the potential publication bias might not be the only cause of the asymmetricity of the funnel plot. Trim and Fill approach still showed a beneficial effect of nicorandil on MACE after imputing 4 assumed missing studies (adjusted OR, 0.61; 95% CI, 0.39 to 0.95) (Fig. 4). So, the impact of potential publication bias is limited.

### Sensitivity analysis

Sensitivity analysis of MACEs demonstrated effect sizes of nicorandil were similar in magnitude and direction to the overall estimate after 1-by-1 exclusion of each individual study (Table 3). Removal of study Kitakaze 2007 [14] or study Pi 2019 [20] could lower the heterogeneity from severe heterogeneity to mild and moderate heterogeneities (Kitakaze 2007: Tau^2^ = 0.05; P = 0.30; I^2^ = 16%; Pi 2019: Tau^2^ = 0.10; P = 0.15; I^2^ = 34%). If both studies were removed, the heterogeneity disappeared (Tau^2^ = 0.00; Chi^2^ = 5.61, P = 0.59; I^2^ = 0%; data not shown), which meant these two studies might be the origin of severe heterogeneity. This result was consistent with the subgroup analysis based on the administration methods of nicorandil, because study Pi 2019 [20] and Kitakaze 2007 [14] had the greatest sample sizes in the intracoronary plus intravenous and the intravenous subgroup respectively.

**Table 3:** Sensitivity analysis of the incidences of MACEs after 1-by-1 exclusion of each individual study

Study removed each time	Statistics after one study removed			Heterogeneity		
	OR	95% CI	*P*	Tau^2^	I^2^ (%)	P
No study removed	0.42	0.27–0.64	< 0.001	0.21	52	0.03
Chen 2015 [6]	0.41	0.26–0.65	< 0.001	0.24	57	0.02
Chen 2020 [23]	0.42	0.26–0.67	< 0.001	0.24	56	0.02
Feng 2019 [5]	0.42	0.26–0.68	< 0.001	0.23	55	0.02
Ishii 2005 [11]	0.37	0.21–0.64	< 0.001	0.35	57	0.02
Kitakaze 2007 [13]	0.37	0.25–0.54	< 0.001	0.05	16	0.30
Lee 2008 [14]	0.40	0.25–0.63	< 0.001	0.23	57	0.02
Ono 2004 [17]	0.43	0.27–0.68	< 0.001	0.22	54	0.03
Ota 2006 [18]	0.44	0.28–0.69	< 0.001	0.21	53	0.03
Pi 2019 [19]	0.50	0.34–0.74	< 0.001	0.10	34	0.15
Qi 2018 [20]	0.43	0.28–0.67	< 0.001	0.20	53	0.03
Wang 2020 [24]	0.42	0.27–0.64	< 0.001	0.21	52	0.03

*CI* confidence interval, *MACE* major adverse cardiovascular events, *OR* odds ratio

## Discussion

Our meta-analysis showed that nicorandil administrations could improve the NRP and complete STR after primary PCI, reduce the incidence of MACEs during in-hospital stay and this improvement could maintain at follow-up. The improved effects on MACEs were mainly driven by reduced incidences of CHF and ventricular arrhythmia (ventricular tachycardia or ventricular fibrillation). Continuous intravenous nicorandil after primary PCI cannot further improve incidence of MACEs. Interestingly the improvement of MACEs could only be detected in studies with intracoronary combined/not combined with intravenous nicorandil administrations, not in studies with only intravenous nicorandil being administrated. Intravenous administration might not be an optimal usage of nicorandil to improve MACEs in patients treated with primary PCI.

The aim of primary PCI is to open the infarct-related artery and salvage more myocardium as soon as possible in patients with STEMI. But restoration of anterograde coronary flow and complete myocardial reperfusion are not always achieved even though there is no residual stenosis, which is known as coronary NRP and is associated with MACEs and poor prognosis. Impaired microvasculature function caused by ischemia or ischemia–reperfusion injury is the main pathophysiology underlying NRP. Nicorandil, as a nicotinamide derivative, can improve both the epicardial coronary artery and microvasculature function via nitrate-like and K^+^_ATP_ agonist effects respectively. Our study convinced the beneficial effects of nicorandil on improvement of NRP (OR, 0.46; 95% CI, 0.36–0.59; P < 0.001) and STR (OR, 2.86; 95% CI, 2.19 to 3.73; P < 0.001).

Several measurements were used for assessing clinical NRP and it is important to be aware of the limitations of these measurements. TIMI flow grade and cTFC are widely used to evaluate the prognosis of primary PCI. Higher TIMI flow grades are associated with improved clinical outcomes and lower mortality [26], but TIMI flow grades cannot reflect tissue perfusion accurately and distal tissue perfusion may vary considerably despite TIMI grade 3 flow is achieved [27]. CTFC attempts to assess the coronary reperfusion more objectively compared with TIMI flow grades. Though cTFC reflects epicardial coronary blood flow velocity accurately, it is not accurate enough to assess the degree of microvasculature injury after primary PCI [28]. While myocardial blush grade (MBG) is used to assess myocardial staining after primary PCI, TMPG assesses myocardial perfusion, based on the evolution (i.e., entry, endurance, and clearance) of contrast media at the myocardial level. In a study by Wong DTL, TMPG had the strongest relationship with coronary microvasculature obstruction (MVO) assessed by cardiac magnetic resonance (CMR) on day 3 post-STEMI, while MBG did not correlate with MVO in patients undergoing primary PCI [29]. So, we chose TMPG as the measurement of choice to accurately evaluate the NRP in our pooled analysis.

Congestive heart failure and ventricular arrhythmia are common complications in patients with AMI due to failed myocardial reperfusion. The improvement of NRP by nicorandil could mitigate the myocardial injury caused by myocardial infarction, which might partially explain the reduced incidences of congestive heart failure and ventricular arrhythmia. There are some other particular effects of nicorandil that might contribute to these benefits.

The predominant electrophysiology effect of high-concentration nicorandil is shortening of the action potential and refractory period [30], which may yield proarrhythmic effects. However, our study showed nicorandil could significantly reduce the incidence of ventricular fibrillation and tachycardia in patients undergoing primary PCI, which is in agreement with some other animal and clinical studies. Study by Hirose et al. [31] demonstrated that nicorandil shortened action potential without increasing dispersion of action potential durations; suppressed the increased dispersion of local conduction velocity during ischemia; increased the size of non-excited area in the epicardial region of the transmural wall (the origin of reentry) by activating sarcolemma K^+^_ATP_ channels, thus preventing ventricular tachycardia during acute global ischemia in arterially perfused canine left ventricular wedges. A historical cohort study by Ueda et al. [32] showed that intravenous nicorandil could reduce the occurrences of ventricular fibrillation and QT dispersion in 83 patients with AMI who underwent successful PCI. QT dispersions in the nicorandil group were shorter than those in the control group 48 h after percutaneous transluminal coronary angioplasty (nicorandil group: 23.2 ± 16.1 ms; control group: 33.4 ± 24.0 ms, P < 0.05). Ventricular fibrillation was observed in 3 patients in the control group, but none in the nicorandil group.

Our study showed that nicorandil could reduce the incidence of heart failure compared with controls in STEMI patients undergoing primary PCI (OR:0.36, 95% CI:0.23–0.57, P < 0.001). Cardiomyocyte apoptosis are crucial events underlying the development of cardiac abnormalities and dysfunction after AMI. Wang S et al. found nicorandil alleviated post-MI cardiac dysfunction and remodeling in left anterior descending coronary artery ligated mice [33]. The mechanisms were associated with enhancing autophagy and inhibiting apoptosis through Mst1 inhibition.

Nicorandil can exert cardiac protections not only by improving the vasculature function but also by enhancing pharmacological preconditioning in cardiac cells and arterioles during ischemic reperfusion. Mitochondrial permeability transition pore (mPTP) opening plays an important role in the myocardial injury during the first minutes after restoration of blood flow [34]. Nicorandil, not only directly but also indirectly via activation of the NO-PKG pathway, opens mitochondrial K^+^_ATP_ channels [35], which is believed to inhibit mPTP opening [36], thus alleviate myocardial injury during reperfusion and enable greater salvage of myocardium.

Yamada et al. [23] used CMR imaging to compare the infarct and edema size in 52 patients with AMI treated by nicorandil with those treated by nitrate. All these patients underwent emergency PCI. The results showed both the edema size on T_2_-weight CMR and the infarct size on delayed enhancement CMR were significantly smaller in patients treated by nicorandil than nitrate (17.7 ± 9.9% vs. 21.9 ± 13.7%, P = 0.03; 10.3 ± 6.0% vs. 12.7 ± 6.9%, P = 0.03, respectively); the presence and amount of microvasculature obstruction were significantly smaller in patients treated by nicorandil than nitrate (39.2% vs. 64.7%, P = 0.03; 2.2 ± 1.3 cm^2^ vs. 3.4 ± 1.5 cm^2^, P = 0.02, respectively).

Antithrombotic therapy plays an important role in the management of patients with AMI. Dual antiplatelet therapy (DAPT) is still recommended in current guidelines for patients undergoing primary PCI [37]. The appropriate use of antithrombotic therapy is vital to effectively balance treatment benefit vs risks and improve outcomes. A recent individual patient level meta-analysis by Valgimigli et al. showed P2Y12 inhibitor monotherapy was associated with a similar risk of death, myocardial infarction, or stroke and a lower bleeding risk compared with DAPT [38]. The effects of nicorandil in P2Y12 inhibitor monotherapy need to be explored in future studies.

There are several limitations in our study.

First, the oral administrations of nicorandil following intravenous or intracoronary nicorandil were reported in only 2 of the included RCTs [13, 16]. Kang et al. reported that 4 weeks oral nicorandil could attenuate sympathetic hyperinnervation after infarction by activating mitochondrial K^+^_ATP_ channels in postinfarcted rat hearts [39]. Whether this beneficial effect can be translated into improvement of clinical prognoses in STEMI patients needs to be further investigated.

Second, there are great heterogeneity in the administration methods of nicorandil, measurements for evaluating NRP, follow-up durations in the studies enrolled. Future large RCTs designed to evaluate the efficacy of nicorandil in different conditions are needed.

Third, subgroup analyses are observational by nature and the possibility of our subgroup analyses being biased by some confounding factors couldn’t be ruled out. So, the results of our subgroup analyses need to be confirmed by future large scale RCTs.

Last, racial differences and genetic polymorphisms may affect efficacy of certain disease processes and medications [40]. All the 18 included trials were performed in Asia (10 in Japan; 7 in China; 1 in Korea). More studies on the cardioprotective effects of nicorandil are expected in countries out of Asia.

## Conclusions

Nicorandil can significantly improve NRP, STR and MACEs in patients with STEMI undergoing primary PCI. The beneficial effects of nicorandil on MACEs might be driven by the improvements of heart failure and ventricular arrhythmia after primary PCI. A combination of intracoronary and intravenous administration might be an optimal usage of nicorandil.

## Supplementary Information

**Additional file 1: Fig. 1**. Risk of bias graph.

**Additional file 2: Fig. 2**. Risk of bias summary.

**Additional file 3: Fig. 3**. Subgroup analysis of major adverse cardiovascular events based on with/without following continuous intravenous nicorandil after primary percutaneous coronary intervene.

**Additional file 4: Fig. 4**. Subgroup analysis comparing major adverse cardiovascular events (MACE) in-hospital stay with MACEs during follow-up after hospital discharge.

**Additional file 5: Fig. 5**. Forest plot of complete ST-elevation resolution.

**Additional file 6: Table 1**. Characteristics of included patients.

## Data Availability

The data and material that support the findings of this study are from published trials available in online database (PubMed, EMBase, and Cochrane Central Register of Controlled Trials).

## Abbreviations

AMI: Acute myocardial infarction
CHF: Congestive heart failure
CMR: Cardiac magnetic resonance
cTFC: Corrected TIMI frame count
MACE: Major adverse cardiac events
MCE: Myocardial contrast echocardiography
mPTP: Mitochondrial permeability transition pore
MVO: Microvasculature obstruction
NRP: No-reflow phenomenon
PCI: Percutaneous coronary intervention
RCTs: Randomized controlled trials
STEMI: ST-segment elevation myocardial infarction
STR: Resolution of ST-segment elevation
TIMI: Thrombolysis in myocardial infarction
TMPG: TIMI myocardial perfusion grade
TVR: Target vessel revascularization
