# Supplementary figures and images for: Clinical outcomes of nicorandil administration in patients with acute ST-segment elevation myocardial infarction undergoing primary percutaneous coronary intervention: a systematic review and meta-analysis of randomized controlled trials
Source: BMC Cardiovasc Disord. 2021 Oct 10;21:488. doi: 10.1186/s12872-021-02301-1 (PMC8504118; doi:10.1186/s12872-021-02301-1)

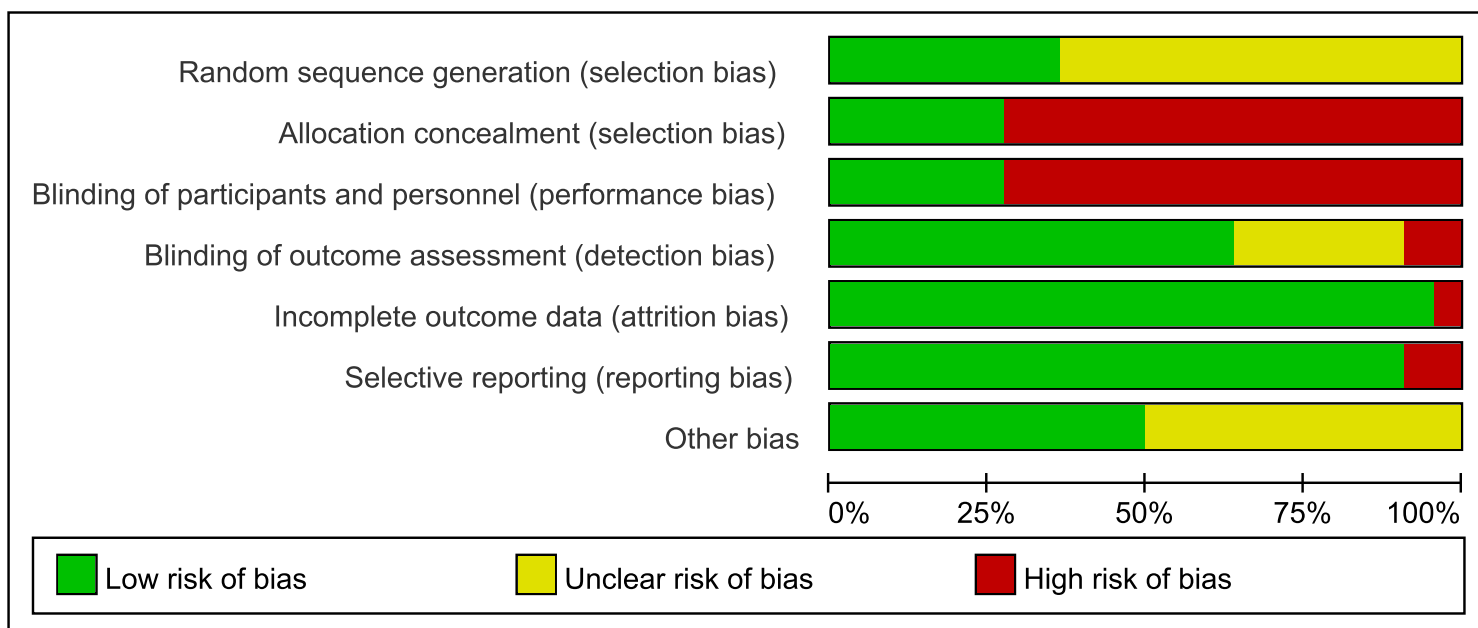

Supplement: Supplementary file 1 — Additional file 1: Fig. 1. Risk of bias graph. [file 12872_2021_2301_MOESM1_ESM.pdf]

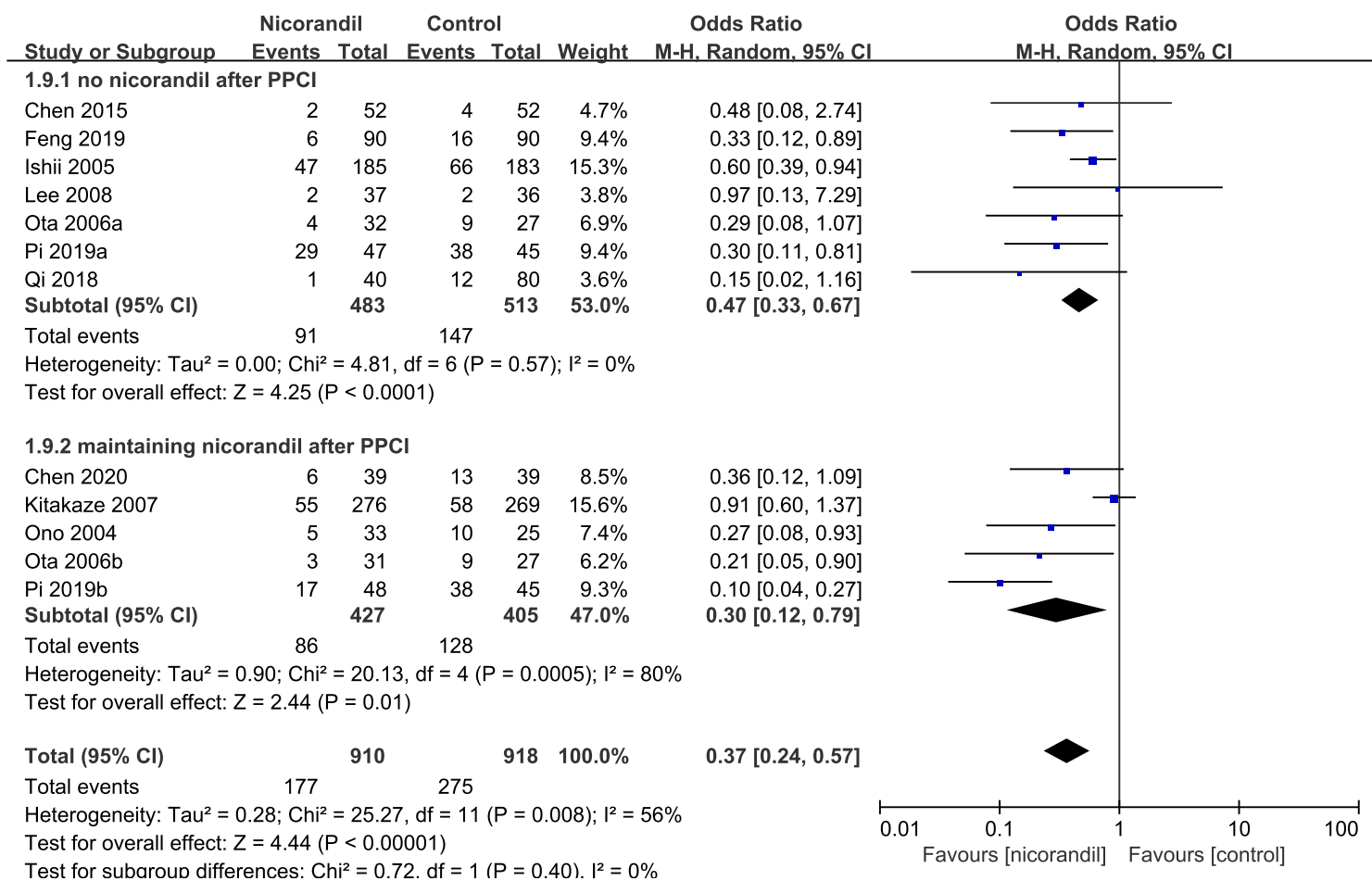

Supplement: Supplementary file 3 — Additional file 3: Fig. 3. Subgroup analysis of major adverse cardiovascular events based on with/without following continuous intravenous nicorandil after primary percutaneous coronary intervene. [file 12872_2021_2301_MOESM3_ESM.pdf]

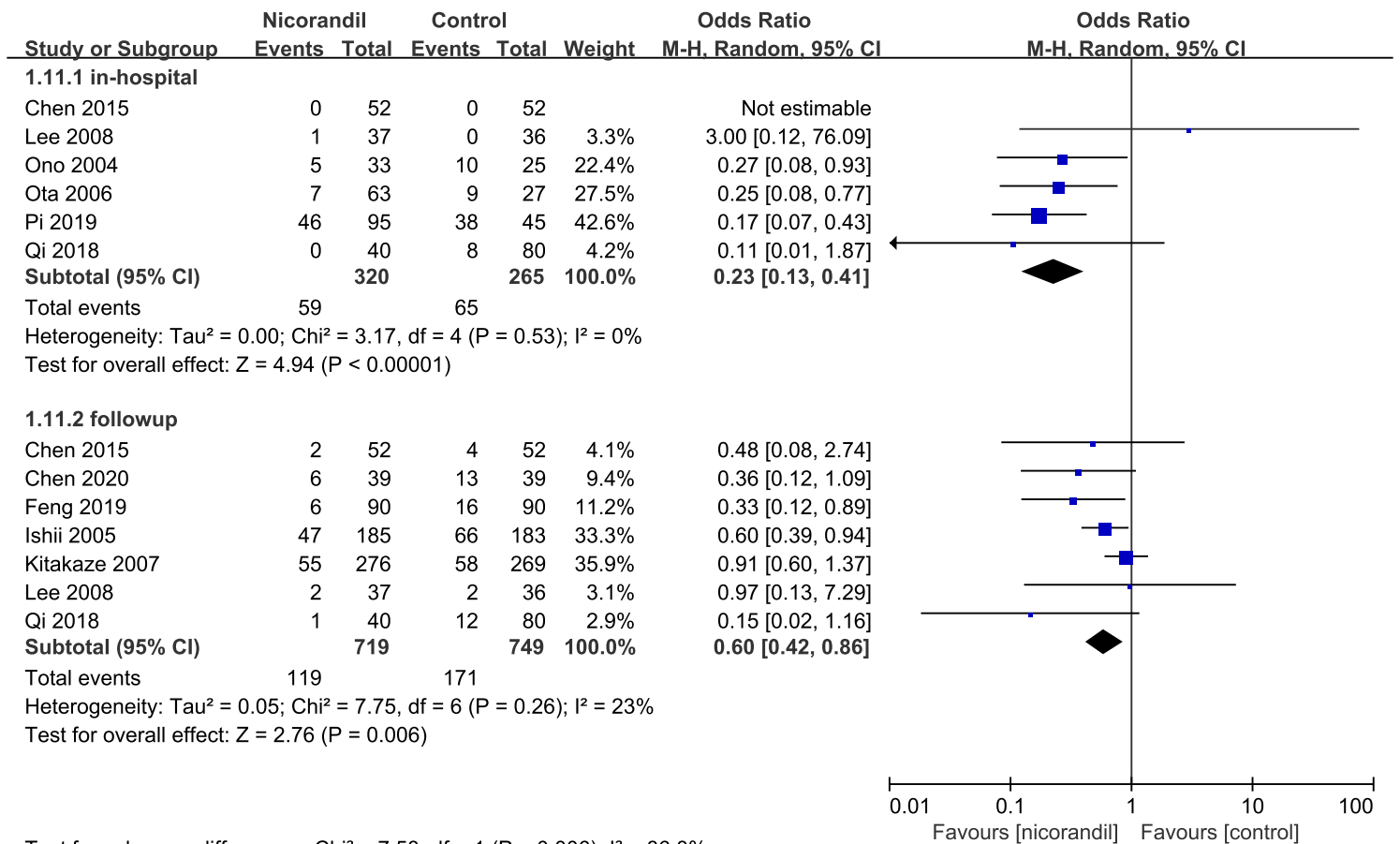

Test for subgroup differences:  $\chi^2 = 7.58$ ,  $df = 1$  ( $P = 0.006$ ).  $I^2 = 86.8\%$

Supplement: Supplementary file 4 — Additional file 4: Fig. 4. Subgroup analysis comparing major adverse cardiovascular events (MACE) in-hospital stay with MACEs during follow-up after hospital discharge. [file 12872_2021_2301_MOESM4_ESM.pdf]

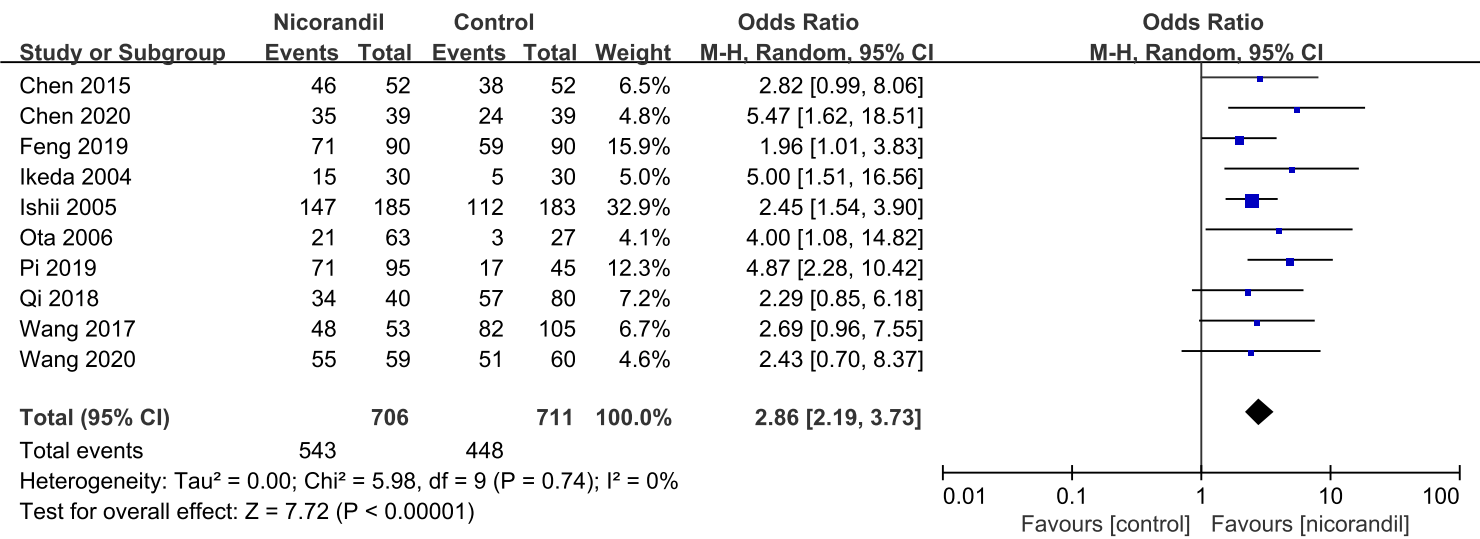

Supplement: Supplementary file 5 — Additional file 5: Fig. 5. Forest plot of complete ST-elevation resolution. [file 12872_2021_2301_MOESM5_ESM.pdf]
