# Supplementary material for: Clinical outcomes of nicorandil administration in patients with acute ST-segment elevation myocardial infarction undergoing primary percutaneous coronary intervention: a systematic review and meta-analysis of randomized controlled trials
Source: BMC Cardiovasc Disord. 2021 Oct 10;21:488. doi: 10.1186/s12872-021-02301-1 (PMC8504118; doi:10.1186/s12872-021-02301-1)
Supplement: Supplementary file 2 — Additional file 2: Fig. 2. Risk of bias summary. [file 12872_2021_2301_MOESM2_ESM.pdf]

	Random sequence generation (selection bias)	Allocation concealment (selection bias)	Blinding of participants and personnel (performance bias)	Blinding of outcome assessment (detection bias)	Incomplete outcome data (attrition bias)	Selective reporting (reporting bias)	Other bias
Chen 2015	?	−	−	+	−	+	?
Chen 2020	+	−	−	−	+	−	+
Feng 2019	+	−	−	?	+	+	+
Fukuzawa 2000	?	−	−	?	+	+	?
Ikeda 2004	?	−	+	?	+	+	?
Ishii 2005	?	+	+	+	+	+	?
Ito 1999	?	−	−	+	+	+	?
Kitakaze 2007	+	+	−	+	+	+	+
Lee 2008	?	−	−	?	+	+	?
Miyazawa 2006	?	−	−	+	+	+	+
Nameki 2004	?	−	−	+	+	+	?
Ono 2004	?	+	−	+	+	+	?
Ota 2006	?	+	−	+	+	+	?
Ota 2006a	?	+	−	+	+	+	?
Ota 2006b	?	+	−	+	+	+	?
Pi 2019	+	−	+	+	+	+	+
Pi 2019a	+	−	+	+	+	+	+
Pi 2019b	+	−	+	+	+	+	+
Qi 2018	?	−	−	?	+	+	+
Wang 2017	+	−	−	?	+	+	+
Wang 2020	+	−	−	−	+	−	+
Yamada 2015	?	−	+	+	+	+	+
