# Supplementary material for: Clinical outcomes of nicorandil administration in patients with acute ST-segment elevation myocardial infarction undergoing primary percutaneous coronary intervention: a systematic review and meta-analysis of randomized controlled trials
Source: BMC Cardiovasc Disord. 2021 Oct 10;21:488. doi: 10.1186/s12872-021-02301-1 (PMC8504118; doi:10.1186/s12872-021-02301-1)
Supplement: Supplementary file 6 — Additional file 6: Table 1. Characteristics of included patients. [file 12872_2021_2301_MOESM6_ESM.docx]

Supplementary table 1 Characteristics of included patients

| Study | Year | Age(years), N/C | Male(%), N/C | DM(%), N/C | Hypertension(%), N/C | Dyslipidemia (%), N/C | Smokers, (%), N/C |
| --- | --- | --- | --- | --- | --- | --- | --- |
| Chen et al [6] | 2015 | 58.1/59.4 | 75.0/69.3 | 26.9/28.9 | 40.4/50.0 | NA | 67.3/63.5 |
| Chen GX et al [23] | 2020 | 67/66.2 | 92.3/89.7 | 30.7/25.6 | 51.3/48.7 | NA | 74.4/76.9 |
| Feng et al [5] | 2019 | 69.2/68.5 | 70.0/72.0 | 42.0/36.0 | 56.0/59.0 | NA | 45/49 |
| Fukuzawa et al [9] | 2000 | 61.5/61.3 | 74.2/71.0 | 32.3/29.0 | 58.1/67.7 | 45.2/48.4 | 61.3/61.3 |
| Ikeda et al [10] | 2004 | 60/63 | 76.7/83.3 | 16.7/20.0 | NA | NA | NA |
| Ishii et al [11] | 2005 | 63/64 | 77.8/84.1 | 33.0/31.7 | 28.1/31.1 | 25.9/28.4 | 35.7/42 |
| Ito et al [12] | 1999 | 60/60 | 80/78 | 25/29 | 53/51 | 27/34 | 60/51 |
| Kitakaze et al [13] | 2007 | 61.1/63.7 | 89.1/81.8 | 39.5/32.9 | 48.5/53.9 | 46.7/46.2 | 68.7/66.1 |
| Lee et al [14] | 2008 | 56.4/60.2 | 83.8/83.3 | 27.0/36.1 | 51.4/66.7 | NA | 86.5/69.3 |
| Miyazawa et al [15] | 2006 | 64/60 | 88/74 | 28/40 | 48/57 | 31/37 | 54/57 |
| Nameki et al [16] | 2004 | 64/62 | 85/85 | 31/26 | 46/44 | 23/44 | 77/78 |
| Ono et al [17] | 2004 | 64/66 | 67/64 | 33/32 | 55/56 | 42/36 | 45/36 |
| Ota et al [18] | 2006 | 62.2/64.2 | 82.5/74.1 | 29/33 | 44/41 | 60/44 | 54/52 |
| Pi et al [19] | 2019 | 68.5/68.7 | 72.6/71.1 | 63.2/62.2 | 62.1/66.7 | 61.1/57.8 | 71.6/71.1 |
| Qi et al [20] | 2018 | 56/60 | 72.5/71.3 | 42.5/33.8 | 55.0/53.8 | 40.0/35.0 | 40.0/46.3 |
| Wang et al [21] | 2017 | 61.6/63.5 | 81.1/82.9 | 24.5/27.6 | 54.7/61.9 | 30.2/37.1 | 56.6/51.4 |
| Wang ZD et al [24] | 2020 | 54/55 | 79.7/78.3 | 10.2/8.3 | 42.4/43.3 | NA | 67.8/68.3 |
| Yamada et al [22] | 2015 | 67/65 | 82/91 | 21/41 | 32/33 | 14/37 | 32/37 |

DM, diabetes mellitus; N/C, nicorandil/control; NA, no answer.
